# Harnessing dendritic cells in inflammatory skin diseases

**DOI:** 10.1016/j.smim.2011.01.006

**Published:** 2011-02

**Authors:** Chung-Ching Chu, Paola Di Meglio, Frank O. Nestle

**Affiliations:** St. John's Institute of Dermatology, King's College London and NIHR Biomedical Research Centre, Guy's and St. Thomas’ Hospitals, 9th floor Tower Wing, Guy's Hospital, London SE1 9RT, United Kingdom

**Keywords:** SALT, skin-associated lymphoid tissue, DCs, dendritic cells, AD, atopic dermatitis, CHS, contact hypersensitivity, DDCs, dermal DCs, MHC, major histocompatibility complex, pDCs, plasmacytoid DCs, TLR, toll-like receptor, SLE, systemic lupus erythematousus, HEL, hen egg lysozyme, iNOS, inducible nitric oxide synthase, TIP-DCs, TNF- and iNOS-producing DCs, NO, Nitric oxide, IDECs, inflammatory dendritic epidermal cells, NR-UVB, narrow-band UVB, PML, progressive multifocal leukoencephalopathy, TCI, transcutaneous immunization, Skin DCs, Homeostasis, Regulatory DCs, Chronic inflammation, Psoriasis

## Abstract

The skin immune system harbors a complex network of dendritic cells (DCs). Recent studies highlight a diverse functional specialization of skin DC subsets. In addition to generating cellular and humoral immunity against pathogens, skin DCs are involved in tolerogenic mechanisms to ensure the maintenance of immune homeostasis, as well as in pathogenesis of chronic inflammation in the skin when excessive immune responses are initiated and unrestrained. Harnessing DCs by directly targeting DC-derived molecules or selectively modulate DC subsets is a convincing strategy to tackle inflammatory skin diseases. In this review we discuss recent advances underlining the functional specialization of skin DCs and discuss the potential implication for future DC-based therapeutic strategies.

## 1 Introduction

The skin is an organ rich in easily assessable immune cells that are ready to provide the first line immune defense mechanisms against a multitude of internal and external pathogen-derived and environmental challenges. As the primary immunological barrier to the external environment, the skin is equipped with a complex network of immune cells, originally described as “skin-associated lymphoid tissue (SALT)” [1] and later as “skin immune system” [2]. Skin immune sentinels consist of both innate and adaptive immune cells [3]. Dendritic cells (DCs), as professional antigen presenting cells, are the main gate-keepers of the immune system, bridging innate and adaptive immunity. It is now becoming clear that DCs with their inherent plasticity can initiate a diverse range of immune responses in addition to conventional functions in the generation of immunity against pathogens [4,5]. In the steady-state, DCs have an essential role in maintaining tissue homeostasis. In pathology, DCs are able to mature into inflammatory DCs at sites of inflammation in both autoimmune and allergic disease, thereby sustaining a continuous activation of the adaptive immune system at sites of inflammation [6].

**Fig. 1:**
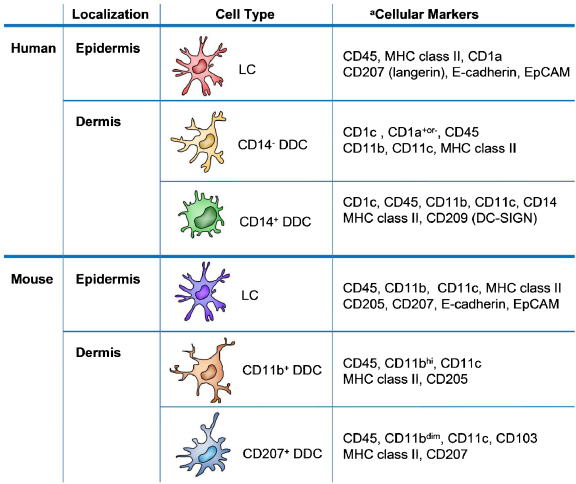
Dendritic cell populations in the skin of human and mice. ^a^Common cellular markers associated with human and mice skin DC subsets.

DCs have a central role in many aspects of skin immunity. Understanding the skin DC network is critically important to tackle skin immune pathology. Over the years much effort has been devoted to characterize skin DC subsets and their functions. Many studies have taken advantage of genetically engineered animal models that allow researchers to selectively examine or target DC subsets under defined conditions. Although breakthrough findings in the murine system have brought valuable insights into our understanding of the skin DC network, the discrepancy between human and murine skin immune systems, in particular the heterogeneity of DCs, donor variability, and diverse markers for DC subsets identification, poses a challenge for translational findings from the murine to the human system (Fig. 1). Different experimental approaches (i.e. *in situ* or *ex vivo* examination; enzymatic digestion or naturally crawl-out methods to isolate skin DCs) applied in various studies have increased the complexity of data analysis. Thus, the functional characteristics of human skin DC subsets are only partially understood.

**Fig. 2:**
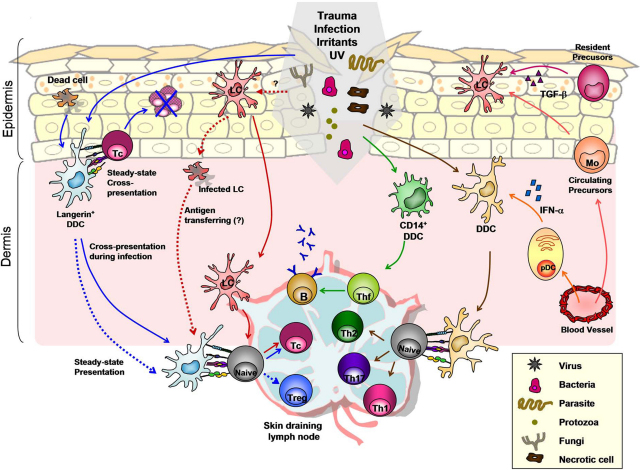
Skin DC subsets and function during tissue homeostasis. Healthy skin consists of a complex network of DCs that play important roles during tissue homeostasis. In the steady state, CD207^+^ DDCs (found in the dermis of mice) can capture dead cells or tissue antigens and promote T cell tolerance via cross-presentation and/or Treg induction in the draining lymph nodes. During tissue damage or infection, LCs and dermal DDCs may initiate cytotoxic T lymphocyte (Tc) responses via cross-presentation. Infected LCs that undergo apoptosis/necrosis could potentially transfer antigens to other cell types capable of cross-presentation. LCs are re-populated by resident or circulating precursors. CD14^+^ DDCs polarize follicular T helper cells (Thf) which promote B cell development, while other DDCs (including CD1a^+^ DDCs) can initiate Th responses which may be sustained by pDCs via IFN-α production. Dotted lines refer to speculative relationships.

In this review we discuss the current understanding of the skin DC network, emphasizing the functional specialization of skin DC subsets in the steady-state conditions (Fig. 2) and pathology, using the common inflammatory skin diseases psoriasis and atopic dermatitis (AD) as examples. This review also highlights current therapeutic approaches as well as new concepts related to harnessing DCs as a convincing immunological intervention for the treatment of chronic inflammatory immune-mediated skin diseases.

## 2 Skin DC subsets and functions

### 2.1 Epidermis (Langerhans cells)

Langerhans cells (LCs) are the primary DC subset in the epidermis of healthy skin. They are radio-resistant and are phenotypically characterized by the expression of langerin (CD207), CD1a, E-cadherin and epithelial-cell adhesion molecule (EpCAM) [7]. Morphologically, they are typified by the presence of Birbeck granules, a tennis racket-shaped cytoplasmic organelle mainly composed by langerin [8]. In the steady state, LCs are situated in the basal and suprabasal layers of epidermis, where they interact with keratinocytes through E-cadherin [9]. E-cadherin ligation may act to maintain LCs in an immature state [10]. Keratinocytes are believed to be an important source of mediators that help to support the development of LCs [11]. In adult quiescent skin, LCs are maintained at a stable density in the epidermis potentially through self-renewal or by skin-resident radio-resistant LC precursor cells [12]. During injury or inflammation when the skin is depleted of resident LCs as in the case of UV-B irradiation, circulating progenitors such as monocytes may enter the inflamed skin and replenish LCs in the epidermis [12–14]. In humans, CD34^+^ hematopoietic progenitor cells [15,16], monocytes [17], as well as dermal CD14^+^ DCs [18] were shown to give rise to LC-like cells *in vitro*. TGF-β is a critical factor for LC development both *in vitro* and *in vivo*, as LCs are absent in the epidermis of TGF-β deficient mice [19].

Despite the abundance of studies in the past decades aiming to uncover the functional role of LCs, the *in vivo* function of LCs is still not fully understood. As one of the first DCs coming into contact with invading pathogens, LCs were believed to have the capacity to sense infection, capture antigens and acquire a strong immunogenic function [20,21]. The classical LC paradigm states that LCs are maintained in an immature state in quiescent skin. Upon encountering pathogens, LCs can capture antigens and undergo a maturation process which involves up-regulation of major histocompatibility complex (MHC) class I and class II molecules, costimulatory molecules including CD40, CD80 and CD86, and chemokine receptors such as CCR7, as well as down-regulation of E-cadherin that allows them to migrate out of the skin to draining lymph nodes, where they present antigens to T cells [22]. Both *ex vivo* isolated LCs or *in vitro* generated LC-like DCs display a strong T-cell stimulatory capacity [23]. In addition, LCs present exogenous antigens to CD8^+^ T cells via the MHC class I pathway, a process referred to as “cross-presentation” and promote a strong cytotoxic T cells responses [23,24].

Early work addressing *in vivo* function of LCs has focused on a mouse model of contact hypersensitivity (CHS) and demonstrated the potential role of LCs in CHS reactions [25]. This however has been challenged as removal of LCs by topical application of steroids [26] or using an inducible or constitutive LC ablation model (LC-deficient mice) showed no difference [27] or even an enhancement in the magnitude of CHS responses [28]. These reports suggested that LCs are dispensable in CHS reactions and raises the possibility that LCs may have an immune inhibitory role. The redundant role of LCs in CHS reaction is proposed to be compensated by a recently characterized subset of dermal resident CD207^+^ DCs. Notably, the concentration of sensitising antigens may be essential for dermal CD207^+^ DC-mediated CHS responses. When only a low concentration of antigens was applied to LC-deficient mice, a diminished CHS reaction was observed potentially due to limited access of antigens by dermal DCs (DDCs) [29,30]. The role of LCs in antimicrobial immunity has also been questioned by the finding that LCs were unable to generate CD8^+^ T cell immunity when the skin was infected with herpes simplex virus [31]. One explanation was that HSV can induce LC apoptosis and therefore diminish their function. It was proposed that apoptotic DCs can be taken up by splenic CD8α^+^ DCs that can efficiently cross-present antigens and initiate anti-viral immunity (Fig. 2). Taken together, the role of LCs in promoting or inhibiting immune responses remains to be fully clarified. It is likely that the nature of antigen as well as the cross-talk involving accessory epidermal cells (i.e. keratinocytes), LCs and DDCs will determined the outcome of the immune responses.

### 2.2 Dermis

#### 2.2.1 Myeloid DCs

Interest on DDCs has significantly increased since a number of early studies functionally characterized resident DCs in the dermis of both murine and human skin [32,33]. Thus far several subpopulations of DDCs have been described under both normal and pathologic conditions. In healthy human skin the majority of DDCs are of myeloid origin and express CD1c (also known as BDCA1) [33], a surface marker widely used to identify the main subset of myeloid DCs in peripheral blood [34]. Zaba et al. extended previous findings and suggested that co-expression of CD1c and CD11c is a useful marker *in situ* to distinguish DDCs from macrophages [35]. These authors further proposed to differentiate between steady-state CD11c^+^CD1c^+^ DDCs and “inflammatory DCs” found in inflamed skin [36]. CD1c^+^ DDCs can be classified into at least three discrete subsets base on their surface expression of CD1a and CD14 [33]. It is now becoming more recognized that distinct subsets of DDCs display differential functional specialization. CD1a^+^CD14^−^ DDCs can be distinguished from CD1a^+^ LCs based on their morphology, phenotype, and transcriptomic profiles [37]. *Ex vivo* isolated CD1a^+^CD14^−^ DDCs have a mature phenotype and are potent inducers of allogeneic naïve CD4^+^ and CD8^+^ T-cell proliferation [23,33,38,39]. In contrast, CD14^+^ DDCs are less mature then CD1a^+^ DDCs and display a reduced capacity to prime naïve T-cell proliferation [40]. Interestingly, IL-23 treatment enhanced the T cell stimulatory capacity of CD14^+^ DDCs. Likewise, IL-23-neutralizing antibody inhibited T cell proliferation stimulated by CD14^−^ DDCs [41]. Although the molecular consequences of IL-23 action on DDCs remain to be investigated, given that DDCs produce IL-23 in skin pathology and the importance of IL-23 in Th17 cell immunity [42], this result may explain at least in part the clinical benefit seen in psoriatic patients treated with anti-IL-12/23p40 (see detail discussion in Section 4.1). While CD1a^+^ DDCs have strong T cell stimulatory capacity, other studies suggest that CD14^+^ DDCs are efficient at antigen up-take, potentially through C-type lectins such as mannose receptor (CD206) and DC-specific ICAM-3-grabbing non-integrin (DC-SIGN/CD209) expressed on the cells surface [18,40]. In addition, Klechevsky et al. demonstrated that CD14^+^ DDCs are able to polarize naïve CD4^+^ T cells into follicular helper T cells, which then promote naïve B cells differentiation [23] (Fig. 2). Although the significance of this finding needs to be further evaluated in more physiological experimental settings, these data underline a previously unappreciated function of CD14^+^ DDCs in the activation of humoral immunity.

Another level of functional specialization for DDCs is highlighted by the detailed characterization of dermal-resident CD207^+^CD103^+^ DCs in mice. This population of DDCs has previously been overlooked, mainly due to its low frequency in the dermis that has led to the assumption that all CD207^+^ DCs found in the dermis are LCs *en route* to draining lymph nodes [43]. A series of animal studies provided clear evidence suggesting that dermal CD207^+^ DCs are distinct from LCs in both their origin and function [44–46]. Recent studies further revealed a unique functional characteristic of CD207^+^CD103^+^ DDCs to cross-present epidermal-derived viral and self antigens to CD8^+^ T cells [47,48]. It appears that CD207^+^CD103^+^ DDCs can capture keratinocyte-derived antigens and cross-present them in skin draining lymph nodes (Fig. 2). Further studies are required to understand how dermal resident CD207^+^CD103^+^ DDCs gain access to epidermal-derived antigens since LCs seem to be dispensable in this process [48].

The unique functional characteristics of CD207^+^CD103^+^ DDCs make this DC subset an attractive target for therapy. This also raises an important question as to the presence of their human DDC counterparts. Dermal CD207^+^ DCs represent less than 5% of DDCs in the human skin (unpublished observation). Whether human CD207^+^ DDCs correspond to the CD207^+^CD103^+^ DDCs described in mice remains to be investigated. Human CD141^+^ (also known as BDCA3) DCs has recently been proposed as putative equivalents of mouse CD8α^+^ DCs, a primary subset of splenic DCs responsible for cross-presentation [49–51]. Despite CD141^+^ DCs representing a minor DC population in peripheral blood, they are found in various non-lymphoid tissues including the skin [34,35,52–55]. The function of CD141^+^ DCs in the human skin is currently unidentified. Future studies are required to address whether skin-resident CD141^+^ DCs have similar functional specialization to those found in the peripheral blood.

#### 2.2.2 Plasmacytoid DCs

Human plasmacytoid DCs (pDCs) represent a minor population of DCs in peripheral blood and are identified in both primary and secondary lymphoid organs including thymus, bone marrow, spleen, and lymph nodes [56,57]. pDCs play a major role in anti-viral immunity because of their extraordinary capacity to rapidly produce large amounts of type I interferons (IFNs) upon viral infection [58,59]. The unique characteristic of pDCs in sensing viral infection is linked to their selective endosomal expression of toll-like receptor (TLR) 7 and TLR9 that, respectively, recognizes single-stranded RNA and DNA derived from pathogens invading cells via the endocytotic route [60].

pDCs are normally absent in the skin and other non-lymphoid tissues in the steady state. However, pDCs can infiltrate the dermis of inflamed skin under several pathogenic conditions, such as psoriasis [61], systemic lupus erythematousus (SLE) [62,63], and certain skin tumors [64]. Several chemotactic factors may be involved in the recruitment of pDCs into peripheral tissues. Chemokine receptor CXCR3 expressed by pDCs can mediate their migration in response to inflammatory chemokines such as CXCL9, CXCL10 and CXCL11 [64]. More recently, chemerin has been shown as a unique inflammatory chemotactic factor that can directly promote pDC migration through interaction with its cognate receptor ChemR23 [65]. Chemerin expression is strongly associated with pDC infiltration in both SLE and psoriasis and therefore may serve as a crucial factor for early pDC recruitment into inflamed skin [65].

During homeostasis, pDCs can sense pathogen-derived nucleic acids but are tolerant to self DNA or RNA released from cells under going necrosis or apoptosis. Breaking tolerance of pDCs to self DNA/RNA can lead to autoimmunity [60]. This process involves the formation of immune complexes between self DNA with DNA-specific antibodies as in the case of SLE [66], or the generation of self DNA and RNA aggregates with the antimicrobial peptide LL-37 as described in psoriasis [67,68]. The pathogenic roles of pDCs in chronic skin inflammatory disorders are discussed in Section 3.1.

### 2.3 Immune regulatory DCs

Several studies have highlighted the critical roles of DCs in regulatory control of immune responses. *In vivo* evidence suggests that DCs can induce “deletional tolerance”, essentially through the presentation of soluble and tissue-associated self antigens to self-reactive T cells in the steady-state [69]. Delivery of hen egg lysozyme (HEL) to DCs in the steady state conditions induces CD4^+^ T cell unresponsiveness to subsequent systemic challenge of HEL. It was proposed that depletion of HEL-specific T cells was a potential tolerance mechanism involved in this model system [70]. Liu et al. further showed that *in vivo* delivery of dying cells to DCs led to immune tolerance. In this model, dying cells were captured and cross-presented to CD8^+^ T cells by the CD8α^+^ DC subset. Following cross-presentation, antigen-reactive CD8^+^ T cells were initially driven into cell cycle but were then deleted [71]. Later work also suggested that the induction of regulatory T cells may be a potential mechanism involved in CD8α^+^ DC-mediated tolerance [72]. These experiments supported the concept that DCs can play a major role in peripheral tolerance by continually capturing and presenting cell-associated antigens or environmental proteins under steady state conditions.

Skin DCs have been shown capable of mediating T cell tolerance. The notion that LCs have immunoregulatory property came from the observation that LC-deficient mice develop enhanced CHS reaction [28]. When transplanting skin graft of a LC-deficient donor to genetically mismatched recipient, efficient graft rejection was routinely observed suggesting that donor LCs are not necessary for graft rejection. Under certain experimental conditions, the role of LCs in supporting long-term skin engraftment was also described [73]. The mechanism of LC-mediated immune regulation is not fully characterized. In the inflamed skin, LCs may produce IL-10 and promote the expansion of regulatory T cells through interaction with receptor activator of NF-κB ligand (RANKL) expressed by keratinocytes [74,75]. In the steady state, DCs of both dermal and epidermal origin were able to transport keratinocyte-associated antigens to skin-draining lymph nodes as demonstrated in transgenic mice expressing membrane-bound form of OVA under the control of the keratin 5 promoter. When adoptively transferring OVA-specific TCR transgenic CD8^+^ T cells (OT-I) into these mice, deletional tolerance was observed as OT-I cells were initially proliferating in skin-draining lymph nodes and then were found disappeared six weeks after transfer. An impaired response of the transferred OT-I cells to subsequent antigenic challenge was also reported in this model [76]. More recently, CD207^+^CD103^+^ DDCs were proposed to be the major skin DC subset involved in antigen transfer and presentation. Future studies are required to further dissect the differential immunoregulatory roles of skin DC subsets in the maintenance of skin homeostasis.

In humans much less is known about regulatory DCs and their *in vivo* functions. Jonuleit et al. and others demonstrated that allogeneic T cells co-cultured with immature DCs became unresponsive to antigenic re-stimulation. In addition, immature DCs induce IL-10 producing regulatory T cells that suppress the function of other effector T cells in a partially IL-10 dependent manner [77,78]. Other work indicated that pro-inflammatory cytokine-matured DCs can efficiently induce regulatory T cells with immune suppressive function [79,80]. The physiological significance of these findings remains to be explored. Thus far, it is not known whether certain subsets of human DCs may have specialized function in immune regulation under tissue homeostasis as described in the murine system.

## 3 Pathogenic role of DCs in inflammatory skin disease

DCs have been recognized as key players in several inflammatory skin diseases. Given their capacity to initiate a cascade of immune responses, DCs of both plasmacytoid and myeloid lineage are thought to display a pathogenic role. Here we focus our discussion on psoriasis and AD, two common types of inflammatory skin pathologies, highlighting the essential role of several DCs subtypes in their respective immunopathogenesis.

### 3.1 Psoriasis

Psoriasis is a common, chronic inflammatory skin disease which affects 2–3% of the Caucasian population and is characterized by sharply demarcated, scaly, red plaques [81]. Psoriasis is believed to result from the combination of environmental triggers and genetic factors, conferring disease susceptibility. Environmental factors include β-haemolytic streptococcal pharyngitis, stress, skin trauma and various drugs (such as β blockers and lithium), while at least nine chromosomal loci with statistically significant linkage to psoriasis have been identified. The major genetic determinant of psoriasis is *PSORS1* located within the major histocompatibility complex (MHC) on chromosome 6p [82], which accounts for up to 50% of the heritability of the disease. Within PSORS1 is the human leukocyte antigen-C (HLA-C) gene which is the strongest candidate gene for psoriasis identified to date, with its allele HLA-Cw*0602 shown to be the predominant risk allele [83,84].

In addition, genome-wide association studies have implicated several other psoriasis associated genes, involved in various biological processes, including IL-23/IL-17 pathway [83,85–89], MHC class I processing [89,90], epidermal cell differentiation [90,91], NF-κB signalling [83,89,92], ubiquitin pathway [90,93], Th2-type response [83] as well as genes of yet unknown function [94].

**Fig. 3:**
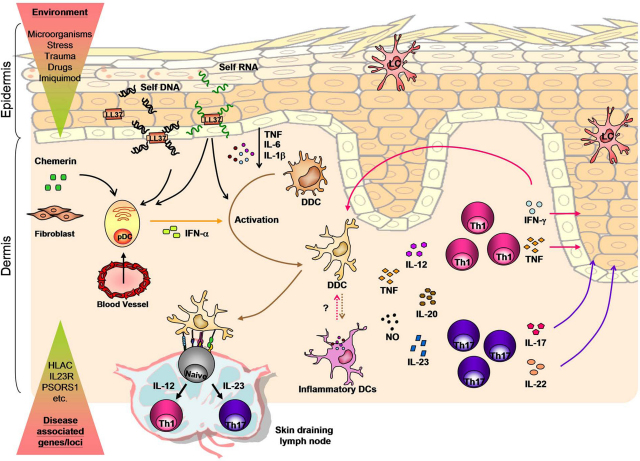
A DC perspective on psoriasis pathogenesis. A combination of environmental triggers and genetic factors leads to disease onset. In the initial phase, pDCs are recruited to pre-psoriatic skin through chemerin and activated by self DNA and/or RNA that is released by keratinocytes and forms complexes with LL-37. IFN-α released by activated pDCs, self-RNA-LL-37 complexes, as well as keratinocyte-derived pro-inflammatory cytokines, TNF, IL-6, and IL-1β, leads to activation of DDCs. Activated DDCs migrate to the skin-draining lymph nodes where they promote the differentiation of naïve T cells into Th1 and/or Th17 cells. In psoriatic lesions, DDCs and inflammatory DCs produce IL-12, TNF, IL-20, nitric oxide (NO) radicals and IL-23 which activate skin resident T cells to produce pro-inflammatory cytokines. Pro-inflammatory Th1 and Th17 cytokines then act on keratinocytes and feedback on DDCs, leading to a sustained and amplified inflammatory status which ultimately leads to the formation of a psoriatic plaque.

Effector cells of both the innate and the adaptive immune system, including keratinocytes, DCs and T cells have all been shown to take part in a dysregulated immune response responsible for the typical histological features of psoriasis (Fig. 3). Excessive keratinocyte proliferation and reduced differentiation lead to thickening of the skin (acanthosis), elongation of the epidermis into the papillary dermis (papillomatosis) and retention of the nucleus in the *stratum corneum* (parakeratosis). Moreover, dermal blood vessels become dilated and increase in number, giving raise to the red colour of psoriasis lesions and are filled with leukocytes that accumulate in both dermis and epidermis. In particular, neutrophils and CD8^+^ T cells are found in the epidermis, while pDCs, myeloid DDCs, and CD4^+^ T cells, mainly Th1 and Th17, are in the dermis.

The initial clinical observation of a case of psoriasis exacerbated by topical treatment with the TLR7 agonist imiquimod [95], the occurrence of an evident IFN-α signature in psoriasis [96] and the presence in elevate number of pDCs in psoriatic skin [95,97] lead to further investigations about the role of pDCs in psoriasis.

pDCs have been identified not only in lesional psoriatic skin but also in uninvolved, healthy-looking psoriatic skin and shown to be the principal IFN-α-producing cells in early and developing psoriasis [61]. This early recruitment of pDCs to the psoriatic skin could be mediated by chemerin abundantly expressed in pre-psoriatic skin adjacent to fully established lesions [65]. Blocking of type I IFN signalling or inhibiting of IFN-α release by pDCs prevented the activation and expansion of pathogenic T cells and the development of psoriasis in a xenograft mouse model of human psoriasis. Moreover, temporal analysis of psoriasis development in this model showed that an IFN-α signalling signature is indeed present in developing psoriasis and precedes the characteristic psoriatic phenotype, including the epidermal changes [61]. Finally, the molecular mechanisms of pDCs activation have been also elucidated. Proteomic analysis of psoriatic skin revealed the pDC activating substance in psoriatic skin. The cathelicidin LL-37, an endogenous antimicrobial peptide produced by keratinocytes and neutrophils and overexpressed in psoriatic skin, has been shown to bind to self-DNA/RNA fragments released by stressed or dying skin cells and to trigger TLR9/TLR7-mediated pDC activation and type I IFN production [67,68]. Additionally, self-RNA-LL-37 complexes signalling through TLR8 have been shown to promote myeloid DC maturation [68]. Therefore, LL-37-mediated breakdown of innate tolerance to self DNA/RNA and a pathogenic cross talk involving stressed epidermal cells and recruited pDC represent one of the first events in psoriasis development, followed by IFN-α-dependent maturation and activation of bystander DDCs and subsequent adaptive immune responses (Fig. 3). Association between self-RNA-LL-37 complexes and mature myeloid DCs observed in psoriatic lesions further suggests a potential pathogenic role of LL-37 in the maintenance phase of psoriasis [68].

While pDCs are believed to be early key players in the upstream events leading to skin lesion formation, myeloid DDCs are thought to be instrumental in sustaining and amplifying the T-cells mediated inflammatory reaction underlying the epidermal hyperplasia.

In the nineties, pioneering early work led to the isolation of DDCs from psoriatic lesions and suggested for the first time that these previously overlooked cells might exert a pivotal role in disease pathogenesis [98]. CD1c and HLA-DR expressing DDCs were found in large number in psoriatic dermis, positioned immediately beneath the hyperplastic epidermis, and surrounded by T cells. DDCs derived from psoriatic lesions were found to be much more effective stimulators of spontaneous autologous T cell proliferation compared with either psoriatic blood-derived or normal healthy skin-derived DC. Moreover, cytokine profiles studies showed that DDCs obtained from psoriatic lesions mediated a T cell response with high levels of IL-2 and IFN-γ, thus contributing to the definition of psoriasis as Th1-type disease [98–100].

Further studies have expanded these findings and DCs have emerged as key cellular players in psoriasis. A 30-fold increase in CD11c^+^ DCs has been shown in the dermis of psoriatic skin lesions compared to uninvolved psoriatic or normal skin [101]. Detailed *in situ* phenotypic analysis of DCs present in psoriatic skin suggested that psoriatic dermis harbors two distinct populations of DCs [36]: a classical CD11c^+^CD1c^+^ population resides normally in the quiescent skin as well as a population of “inflammatory DCs” identified by their CD11c^+^CD1c^−^ phenotype [102].

The CD11c^+^CD1c^−^ inflammatory DCs are relatively immature DCs, with little dendritic cell-lysosomal-membrane-associated protein (DC-LAMP) and DEC-205/CD205 co-expression and decreased HLA-DR expression while showing some expression of CD14^+^, DC-SIGN, and CD163 [102]. Inflammatory DCs are also quite heterogeneous in their function, as they possibly include TNF- and inducible nitric oxide synthase (iNOS)-producing DCs (named TIP-DCs), IL-20-producing DCs and IL-23-producing DCs.

TIP-DCs were first described in the spleen during a murine model of Listeria monocytogenes infection [103] and have also been found in murine *Escherichia coli* [104], *Brucella melitensis* [105] and influenza infection [106] where they are important for pathogens clearance. In humans, iNOS and TNF positive CD11^+^ DCs are abundant in psoriatic skin lesions and are reduced following successful psoriasis treatment [107]. Both TNF and iNOS are well established pro-inflammatory mediators in psoriasis. TNF induces expression of intracellular adhesion molecule-1 (ICAM-1), neutrophils-chemoattractant IL-8 as well as pro-inflammatory cytokines, IL-6 and IL-1 in keratinocytes. Nitric oxide (NO) generated from iNOS in inflamed tissues induces vasodilation and inflammation, thus possibly accounting for the vasodilation of dermal blood vessels in psoriatic skin. Other pro-inflammatory mediator produced by “inflammatory DCs” included IL-20 [108], which enhances keratinocytes activation and proliferation, and, very importantly, IL-23 [101,109].

In just ten years from its discovery [110] IL-23 has quickly emerged as a key pro-inflammatory molecule in psoriasis. IL-23 is highly expressed in psoriatic skin lesions and is down regulated following successful conventional and biologics systemic therapies [111,112]. We and others have shown by genome-wide association studies that several genes of the IL-23 pathway, including *IL23R*, *IL12B* and *IL23A* are associated with psoriasis [83,85,86]. Intradermal injection of IL-23 in mice leads to erythema, induration and prominent dermal papillary blood vessels with histopathological features resembling psoriasis [113]. We have recently shown that selectively targeting IL-23 prevents psoriasis development in a clinically relevant psoriasis mouse model [114]. Moreover, IL-23 plays a role in terminal differentiation and peripheral pathogenicity of effector Th17 cells that infiltrate psoriatic lesion [115] where high level of pro-inflammatory Th17 cytokines, including IL-17A, have been detected [116].

DDCs are the main producers of IL-23 in psoriasis [101,109,117,118]. The crucial link between DDCs and the IL-23/Th17 axis has been further strengthen by *ex vivo* studies showing that DDCs obtained from psoriatic lesions, but not those obtained from normal skin, activates T cells to produce both IL-17 and IFN-γ [102]. Finally, DDCs are also a potential source of the Th1 polarizing cytokine IL-12, although there are conflicting reports in the literature about weather or not there are higher level of IL-12 mRNA and protein in psoriatic *vs.* normal skin [109,119,120].

Taken together, IL-23 and IL-12 secreted by DDCs induce activation of skin resident T cells with release of pro-inflammatory Th1 and Th17 cytokines, that act on keratinocytes, which in turn sustain and amplify the chronic inflammatory disease process by producing pro-inflammatory cytokines, chemokines, members of the S100 family and antimicrobial peptides (e.g. TNF, IL-8, CCL-20, S100 molecules, defensins, cathelicidins) (Fig. 3).

Therefore, a critical role for skin DCs as both direct and indirect sources of pro-inflammatory mediators, complementing their classic role as antigen-presenting cells, is being recognized in psoriasis. However it remains to be established weather CD11c^+^CD1c^+^ “resident DCs” and CD11c^+^CD1c^−^ “inflammatory DCs” are effectively two distinct populations of different origin and function or “inflammatory DCs” are rather “resident DCs” in an activated status. A very recent study has started to shed some light on this topic, showing that the transcriptome of psoriatic CD11c^+^CD1c^−^ inflammatory DCs is closely related to that of CD11c^+^CD1c^+^ resident DCs [121]. Nevertheless, psoriatic CD11c^+^CD1c^−^ inflammatory DCs expressed a wide range of inflammatory molecules, including TNF-related apoptosis-inducing ligand (TRAIL) and several other inflammatory mediators, compared to skin-resident CD11c^+^CD1c^+^ DCs. It has been suggested that CD11c^+^ myeloid DDCs are capable of remarkable plasticity and may differentiate into inflammatory DCs, which encompass both TIP-DC in psoriasis and IDECs present in AD (see below), depending on environmental triggers [122,123].

Further studies, perhaps involving humanized mouse models of psoriasis, will be needed to clarify the exact nature of inflammatory DCs in skin inflammation.

### 3.2 Atopic dermatitis

**Fig. 4:**
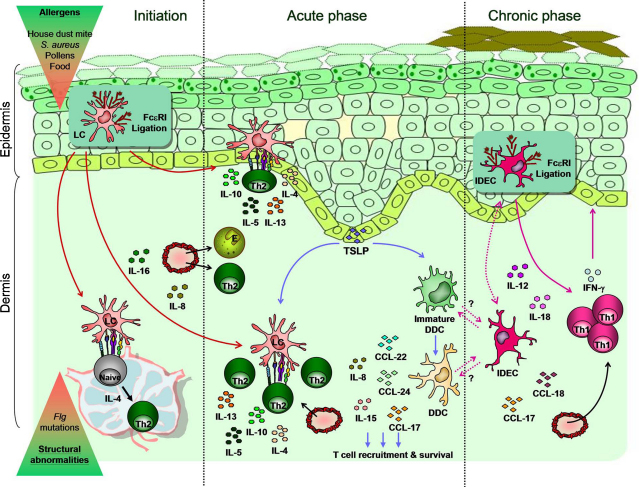
Pathogenic roles of skin DC subsets in atopic dermatitis. In atopic dermatitis (AD), genetically determined epidermal-barrier dysfunction allows the penetration in the skin of allergens that trigger an inflammatory response sustained by cells of the innate and adaptive immunity. In the initial phase of the disease, FcɛRI ligation induces epidermal LCs to produce eosinophils and Th2 cells recruiting-cytokines IL-8 and IL-16 and to migrate to local skin-draining lymph nodes where they promote polarization of Th2 cells producing IL-4, IL-5, IL-10 and IL-13. In the acute phase of the disease, characterized by eczematous plaques and spongiosis, activated LC can induce Th2 response *in situ* in the skin, possibly both in the epidermis and in the dermis, the latter being favored by thymic stromal lymphopoietin (TSLP) produced by keratinocytes. TSLP also promotes DDC antigen-independent maturation and production of pro-inflammatory cytokines and chemokines (CCL-17, CCL-24, CCL-22, IL-8, IL-15) enhancing T cell recruitment and survival in the skin. In the chronic phase of AD, characterized by increased epidermal thickness and plaques lichenification, FcɛRI ligation of IDECs promotes production of IL-12 and IL-18, thus inducing a switch toward a Th1-polarizing environment with Th1 cells secreting IFN-γ. IDEC producing T cell recruiting-chemokines CCL-17 and CCL-18 have been also recently identified in the dermis, raising the possibility that they might migrate from/to the epidermis or are generated from immature and/or activated DDCs.

Atopic dermatitis (AD), also named atopic eczema, is a chronic relapsing, inflammatory skin disease often associated with other systemic atopic disorders such as asthma, food allergy, and allergic rhinitis. Acute AD is characterized by eczematous patches and plaques, epidermal edema (spongiosis) and prominent cellular infiltrate in the dermis. In subacute and chronic phases plaques are lichenified, the epidermis is thickened and its upper layer becomes hypertrophied [124]. Recent insights into the phatophysiology of AD point to an important role for genetically determined structural abnormalities in the epidermis combined with immune dysregulation. Genetic variants of filaggrin, which contribute to the keratin cytoskeleton, have been identified in AD [125,126]. Such epidermal-barrier dysfunction allows the penetration in the skin of allergens such as pollens, house-dust-mite products, microbes and food allergens that trigger an overwhelming inflammatory response mediated by cells of both innate and adaptive immunity. Although AD has been considered a paradigmatic Th2-mediated disease, sequential biopsies in AD patients after exposure to aeroallergens have demonstrated a biphasic immunologic response characterized by a switch toward a Th1 phenotype in later and more chronic phases of the disease [127] (Fig. 4). Two different populations of skin myeloid DCs seem to play a pivotal role in these two phases of AD: LCs and inflammatory dendritic epidermal cells (IDECs) [128].

LCs in AD lesions have been shown to display a high-density of IgE high-affinity receptor (FcɛRI) [129]. This allows them to bind to, internalize and present allergens specific for the IgE molecules, ultimately inducing a typical Th2 type response, characterized by increased IgE production and induction of Th2 cells producing IL-4, IL-10, and IL-13 [130] either in the local skin-draining lymph nodes or possibly *in situ* in the skin. Moreover, *in vitro* studies suggest that on ligation of FcɛRI by IgE, LCs produce pro-inflammatory cytokines IL-8, MCP-1/CCL-2 and IL-16 [130] thus favoring the recruitment to the skin of eosinophils, monocytes and T cells, respectively. Additionally, LCs express high levels of the receptor for thymic stromal lymphopoietin (TSLP), an IL-7-like cytokine, which is abundantly produced by AD keratinocytes [122,131]. Interestingly, high levels of TSLP in AD lesions correlates with increased CD207^+^ DCs redistribution from epidermis to the dermis where they display a mature phenotype (DC-LAMP^+^), thus suggesting that TSLP receptor^+^ DCs in the dermis may be mature LCs [131]. TSLP is known to induce Ag-independent maturation of DC, to maintain their survival and to induce production of CCL-24/Eotaxin2, CCL-17/TARC and CCL-22/MDC as well as IL-8 and IL-15, which favor recruitment of Th2 cells toward inflammatory sites [132]. Finally, TSLP conditions DCs to promote Th2 cells, which produce typical Th2 cytokines, IL-4, IL-5, IL-13, as well as TNF [133].

IDECs were first described using flow cytometric analysis of epidermal single-cell suspensions of AD lesions [128]. Following their discovery IDECs were defined by the surface phenotype of CD11c^+^, HLA-DR^+^, Lin^−^, CD1a^+^, CD206^+^, CD36^+^, FcɛRI^high^, IgE^+^, CD1b/c^+^, CD11b^+^, and DC-SIGN^+^ [128,134].

In contrast to what is described for LCs, *in vitro* FcɛRI ligation of IDECs results in production of IL-1, MIP-1α, IL-16, and Th1-polarizing cytokines IL-12p70 and IL-18 leading to IFN-γ production [130]. Therefore, stimulation of these DCs by allergens via FcɛRI may be responsible for the chronic maintenance phase of AD sustained by a pronounced Th1 cytokine profile.

Recently, a discrete population bearing IDEC markers has been found in the dermis of AD lesions [122] and it has been suggested to re-define IDECs as part of the myeloid inflammatory DC populations spanning both dermal and epidermal compartments of inflamed tissues [122,123]. In contrast to inflammatory DCs present in psoriasis, those found in AD do not express iNOS but are possibly responsible for the high level of CCL17 and CCL18 found in AD [122].

## 4 Clinical significance of DCs as therapeutic target during skin inflammation

**Fig. 5:**
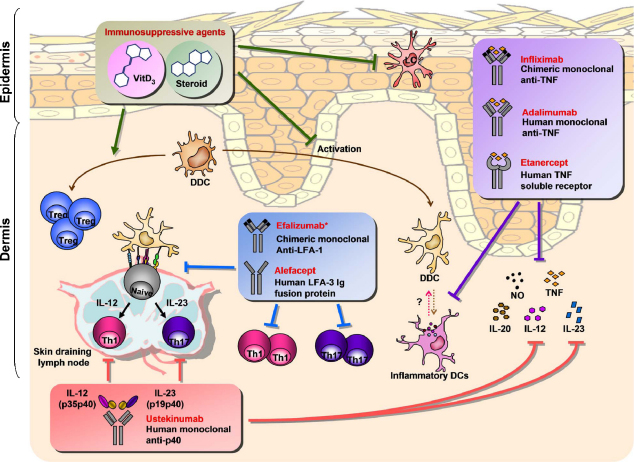
Harnessing skin DCs for therapeutic intervention in psoriasis. Effective targeting of the psoriatic cytokine network with TNF neutralizing antibodies or soluble receptors reduces DDC numbers, interferes with DDC activation, and inhibits down-stream effector molecules. Targeting the common IL-12/IL-23p40 subunit reduces the production of DC-derived pro-inflammatory cytokines and impairs Th1 and Th17 cell responses. Immunosuppressive agents may interfere with LC distribution, DDC activation, and promote the generation of regulatory T cells. Finally, targeting LFA-1 blocks T cell migration and interaction with DCs, while human LFA-3 Ig fusion protein binds to CD2-expressing cells and interferes with DC-T cell interaction. *Efalizumab has recently been withdrawn from the market due to its side effects.

Harnessing DCs is a sensible therapeutic intervention due to their diverse pathogenic roles in inflammatory skin diseases. Clinical applications are being developed to directly target DC-derived molecules or manipulate DC functions. Current approaches and new concepts related to targeting DCs in skin pathology is summarized in Fig. 5.

### 4.1 Anti-cytokine therapy

Given the fundamental role of DCs in shaping immune inflammatory responses in skin it is not surprising that the clinical benefit of several conventional anti-psoriasis therapies, including psoralen and ultraviolet A (PUVA) [135], narrow-band ultraviolet B (NR-UVB) [111,136,137] as well as cyclosporine therapy [22] correlates with a significant decrease in DDC numbers and/or the expression of DC-derived proinflammatory mediators iNOS, TNF, IL12/23p40 and IL-23p19 in psoriatic skin. Similarly, the effective targeting of the psoriatic cytokine network [138] with either neutralizing antibodies or soluble receptors has also been shown to impact on DC biology. Currently regarded as the gold standard treatment for patients with moderate-to-severe psoriasis, anti-TNF strategy is achieved by using either a human p75 TNF-receptor Fc fusion protein (etanercept), a humanized chimeric anti-TNF monoclonal antibody (infliximab), or a fully human monoclonal anti-TNF antibody (adalimumab). Etanercept treatment has been shown to reduce DDC numbers and downstream effector molecules such as iNOS, IL-20, IL-12/23p40 and IL-23p19 [112,139]. Moreover, DDCs rapidly down-regulated co-stimulatory and maturation markers CD83, DC-LAMP, CD86, and HLA-DR after 2 weeks of etanercept treatment [139]. Consistently, etanercept blocked *in vitro*–derived DC maturation, IL-23 production and immunostimulatory capacity, and shifted differentiation toward a macrophage-like phenotype [139]. Recently, analysis of genomic profiles of etanercept-treated patients showed that myeloid cells-expressed genes were the most rapidly down-modulated by etanercept [140], suggesting that TNF inhibition directly modulates myeloid-lineage gene products, which in turn affect T cells activation. Interestingly expression of CD1c and CD207 was on the contrary unregulated by etanercept treatment, suggesting that either resident CD1c^+^ DDCs or LCs do not play an active role in TNF-driven inflammation or they have a rather beneficial role [140]. Although much less investigated, similar effects in terms of DDC numbers and downregulated maturation markers have been reported for adalimimab [141], while infliximab has been shown to affect the *in vitro* differentiation of monocyte-derived DCs and their antigen-presenting capacity [142]. Collectively these data suggest that the clinical benefit of TNF neutralization in psoriasis might derive from the attenuation of inflammatory DC activity in the lesions. This is not surprising given the dual role of TNF as critical inducer of DC maturation and activation as well as proinflammatory mediator for T cells activation.

Another therapeutic strategy that interferes with DC activity involves targeting IL-23 [42]. Two anti IL-12/IL-23p40 monoclonal antibodies (mAbs) have been developed, namely ustekinumab (formerly CNTO-1275) and briakinumab (formerly ABT-874). Both ustekinumab and briakinumab are human IgG1 mAbs that bind to the p40 subunit of human IL-12 and IL-23, and prevent its interaction with IL-12Rb1. While briakinumab is currently in phase III clinical trials [143], ustekinumab has been recently approved for moderate-to-severe psoriasis on the basis of its efficacy in 70–80% of cases [144,145]. Designed in the late 1990s with the aim to block IL-12 (IL-23 was still unknown at that time), ustekinumab targets the common IL-12/23p40 subunit, thus blocking both IL-12 and IL-23 signalling. Early study evaluating the effect of ustekinumab at cellular and molecular level showed a decrease in lesional CD11c^+^ DCs at 2 wk, although this decrease was not statistically significant [146]. Of importance, mRNA levels of IL-12/IL-23p40, IL-23p19 and TNF, were all significantly decreased in the responder populations [146], suggesting that neutralization of IL-12/IL-23 reduces the production of DC-derived pro-inflammatory cytokines. Although preliminary, these results open the possibility that the beneficial effect of targeting IL-12/IL-23 might derive from both an impaired Th17 response but also from the interruption of an autocrine feedback of IL-12/IL-23 on DCs.

Further studies, aiming to dissect the molecular and cellular events following anti-IL-12/IL-23 mAbs administration to large cohort of patients are needed to fully understand the mechanisms of action of this promising new therapeutic agent.

Although referred as anti-T cells therapy, two other biologics drugs for the treatment of moderate-to-severe psoriasis interfere with the interactions between antigen-presenting cells and T-cells at the level of the immunological synapse and are therefore briefly mentioned here.

Alefacept, a CD2-binding leukocyte-function associated antigen (LFA)-3 Ig fusion protein has been proposed to clear psoriasis lesions through the depletion of CD2-expressing cells [147], which includes T cells but also DCs, although to low level. Interestingly, DC-derived pro-inflammatory molecules iNOS and IL-23 are also reduced suggesting that although T cells are the primary target, alefacept also impairs DC activation [147]. Efalizumab, a human mAb directed against LFA-1 which blocks the interaction between LFA-1 on T cells and ICAM-1 on DCs, has been also shown to be effective in clearing psoriatic lesions as well as in reducing CD11c^+^ DC numbers [107]. However, efalizumab was recently withdrawn from the Europe and US market after 5 years of use because of 3 cases of progressive multifocal leukoencephalopathy (PML), a serious life-threatening infection [148].

Taken together data coming from successful psoriasis treatment not only provides further support for the key role of DDCs in the pathogenesis of psoriasis and AD but also suggests that DC can be a useful therapeutic target for the treatment of other immune-mediated dermatological conditions.

### 4.2 Generation of immune regulatory DCs

It is now becoming clear that differentially modulated DCs can become tolerogenic and acquire an immunoregulatory capacity. Several studies reported that anti-inflammatory or immunosuppressive biological and pharmacological agents are able to induce regulatory DCs [149]. Notable examples are gucocorticoids, mycophenolate mofetail, rapamycin (sirolimus), and acetylsalicylic acid (Aspirin) [150]. DCs interacting with regulatory T cells may also acquire immune regulatory properties [151]. Common features of DCs that are affected by immunosuppressive agents include decreased antigen uptake and processing [152], reduced maturation phenotype and function [153], enhanced IL-10 production [154,155], and enhanced DC apoptosis [154,156,157]. In addition, several studies have shown that DCs manipulated to acquire regulatory properties are able to promote the expansion of regulatory T cells [153,158]. Upregulated expression of inhibitory receptors immunoglobulin-like transcript (ILT) 3 and ILT4 is observed in almost all selectively conditioned DCs [159] and is proposed to be involved in the function of regulatory DCs [151,160]. Signalling through ILT3 inhibits NF-κB activation and the expression of costimulatory molecules in antigen presenting cells, which then lead to reduced T-cell priming capacity [151]. When acting on T cells, ILT3 is able to promote CD4^+^ T cell anergy and prevent the generation of cytotoxic activity of CD8^+^ T cells [160]. Expression of ILT3 and ILT4 may serve as markers to identify regulatory DCs, although their expression has been shown dispensable for their regulatory T cell-inducing capacity [161].

The use of immunomodulatory agents has been reported in the treatment of chronic inflammatory skin diseases. Among several agents investigated, the active form of vitamin D_3_ (1α,25-dihydroxy D_3_) and its analogues, alone or in combination with corticosteroids, have been shown to be useful drugs for the treatment of mild to moderate form of psoriasis due to both their effectiveness and high safety profiles [162,163]. Traditionally, the beneficial effects of vitamin D_3_ and its analogues were believed to be linked to their capacity to inhibit differentiation and induce apoptosis on keratinocytes. Later studies revealing the profound regulatory effects of vitamin D_3_ and its analogues on the phenotype and function of DCs have brought new insights into potential mechanisms of vitamin D_3_ treatment in psoriasis. Several studies demonstrated that vitamin D_3_-treated DCs were resistant to maturation and produced diminished amounts of IL-12 and IL-23 as well as reduced T cell-stimulatory capacity [164]. In the epidermis, vitamin D_3_ was also reported to impair LC distribution and antigen-presentation property [165,166]. Vitamin D_3_-induced RANKL expression on keratinocytes may promote IL-10 production by LCs and the induction of regulatory T cells [74]. Psoriasis lesions treated with calcipotriol, a vitamin D analogue, showed up-regulated ILT3 expression on DCs [161]. Overall, generation of regulatory DCs could be a useful approach for DC-mediated inflammatory skin diseases.

### 4.3 Antigen delivery to skin DCs

The skin has long been appreciated as a potential site for antigen delivery since it is rich in easily assessable DCs. Topical application of both peptides and full proteins following barrier disruption (e.g. tape stripping) and with the aid of appropriate adjuvants (e.g. CpG or cholera toxin), an approach known as transcutaneous immunization (TCI), has been shown able to promote systemic cellular and humoral immune responses [167]. Both LCs and DDCs are believed to be involved although the relative contribution of each skin DC subset is not yet fully understood. A more specific approach to selectively deliver antigens to DCs has been proposed using antigens conjugated with antibodies against endocytic receptors expressed by DCs [168]. Pioneer studies using OVA antigen conjugated with antibody against DEC-205, an endocytic receptor abundantly expressed by lymphoid DCs, has demonstrated an efficient antigen uptake by DCs comparing to the un-conjugated OVA protein [169]. Co-administration of DEC-205-conjugated antigens with potent DC maturation stimuli has led to a strong CD4^+^ and CD8^+^ T cells immunity [170]. These findings have recently been extended to target different DC subsets via a variety of DC receptors with the aim to generate potent anti-tumor immunity [168]. Notably, when antigen delivery to DCs was conducted in the steady state (without DC maturation), antigen-specific tolerance instead of immunity was induced potentially through the depletion of effector T cells [70,169] as well as the induction of regulatory T cells [72,171]. This approach have also been shown to prevent the onset and progression of type I diabetics in mouse models [172,173], as well as to induce long-term allogeneic skin graft survival in a transplantation model [174], where immune tolerance induction is required.

Although antigen delivery to DCs has great therapeutic potential, several questions remain to be addressed before applying this to the treatment of inflammatory skin diseases. For example, in the context of chronic skin inflammation, inadvertent DC maturation during antigen targeting could be avoided by co-administration of immune regulatory agents such as vitamin D_3_ and steroids. Indeed, UV-B irradiation or vitamin D_3_ analogue treatment prior to TCI has been shown to induce regulatory T cells and immune tolerance even in the presence of strong DC maturation stimuli [175,176]. Exposure to vitamin D_3_ during tolerance induction may also “imprint” a skin homing phenotype on regulatory T cells [177] and thus help to retain their immune suppressive function in the skin. Another obstacle is the lack of defined autoantigens in many inflammatory skin diseases including psoriasis. The discovery of putative autoantigens would dramatically enhance the specificity of current available therapies. Finally, it is essential to further improve our knowledge of skin DCs subsets and their functional characteristics, in particular translating the current understanding from the murine system to the humans. This will provide the foundation for future therapeutic studies to selectively target a certain subset of skin DCs aiming to generate a specific type of immune response.

## 5 Conclusions

DCs with their inherent plasticity play crucial roles in initiating and modulating immune responses. Recent advance in the knowledge of skin DCs network in health and pathology has opened the avenues for the development of therapies that harness skin DCs with specialized properties to control immunity. Thus far, several therapeutic interventions targeting skin DCs have been proven beneficial to psoriatic patients. Future studies are required to further understand the contribution of skin DC subsets in immunity and tolerance, as well as the cross-talk among the complex network of DCs subsets, skin-resident innate and adaptive immune sentinels, and accessory epidermal and dermal components during homeostasis and pathology. This will provide significant insights in future translational research studies and ultimately lead to the development of novel therapies.
